# Supplementary material for: Multivariate genome-wide association study of leaf shape in a Populus deltoides and P. simonii F1 pedigree
Source: PLoS One. 2021 Oct 28;16(10):e0259278. doi: 10.1371/journal.pone.0259278 (PMC8553126; doi:10.1371/journal.pone.0259278)
Supplement: S1 Fig — Histograms with probability density curves (red) of normal distributions for each univariate trait of L (A), W (B), W31 (C), W21 (D), W32 (E), A (F), and the ratio of L to W (G) in the randomized complete block design derived from the F1 progeny of Populus deltoides × Populus simonii. (DOCX) [file pone.0259278.s001.docx]

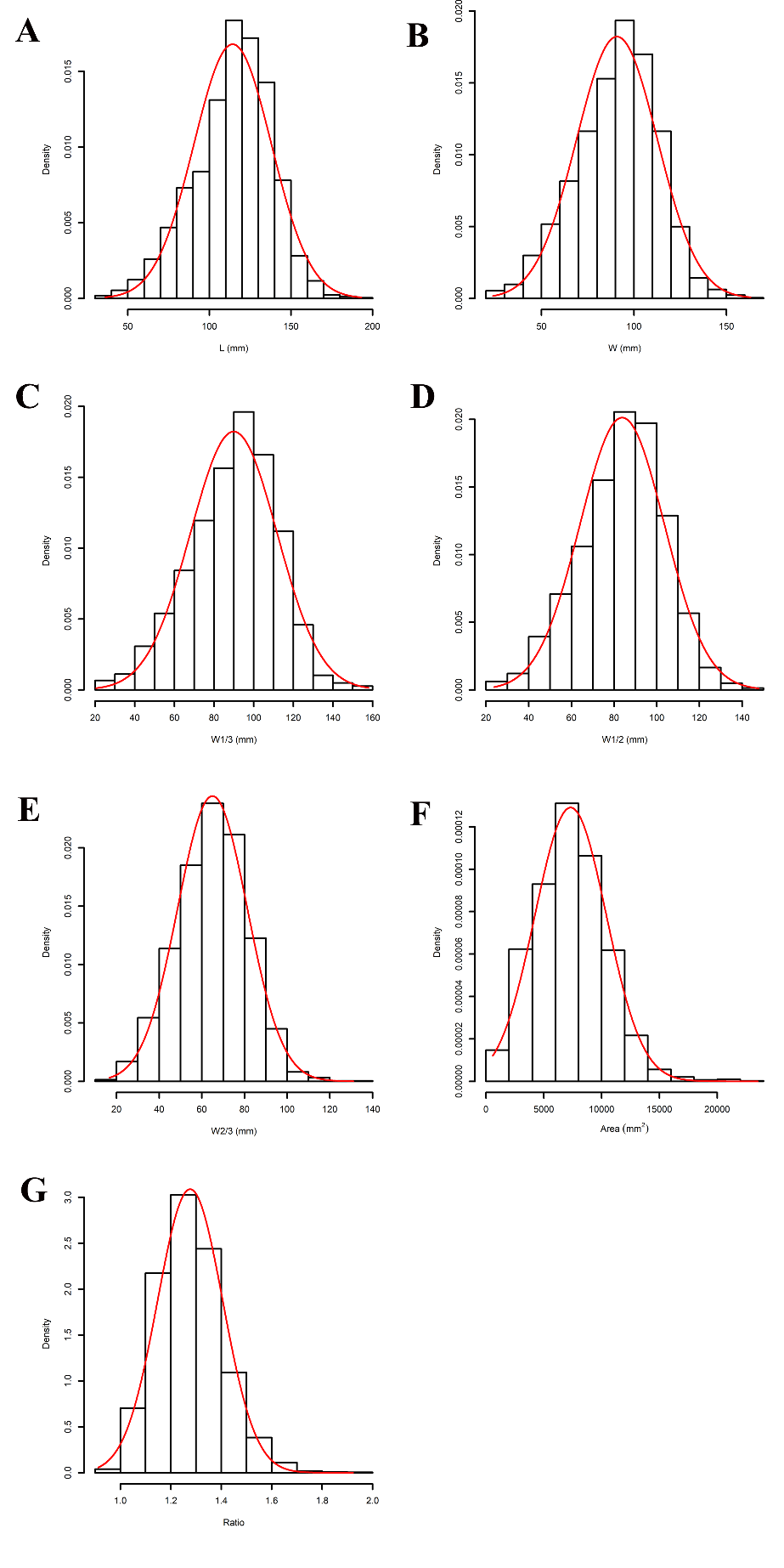


**S1 Fig.** Histograms with probability density curves (red) of normal distributions for each univariate trait of L (A), W (B), W31 (C), W21 (D), W32 (E), A (F), and the ratio of L to W (G) in the randomized complete block design derived from the F1 progeny of *Populus deltoides* $\times$ *Populus simonii*.
