# Supplementary material for: Multivariate genome-wide association study of leaf shape in a Populus deltoides and P. simonii F1 pedigree
Source: PLoS One. 2021 Oct 28;16(10):e0259278. doi: 10.1371/journal.pone.0259278 (PMC8553126; doi:10.1371/journal.pone.0259278)
Supplement: S2 Fig — Manhattan plots of the association analyses without genomic control for each univariate trait of L (A), W (B), W31 (C), W21 (D), W32 (E), A (F), and the ratio of L to W (G) across the 19 chromosomes of the reference genome of P. trichocarpa. The horizontal dashed line indicates the genome-wide significant threshold of 5.33, a base 10 logarithm of p-value based on the Bonferroni correction at the 0.05 significant level. (DOCX) [file pone.0259278.s002.docx]

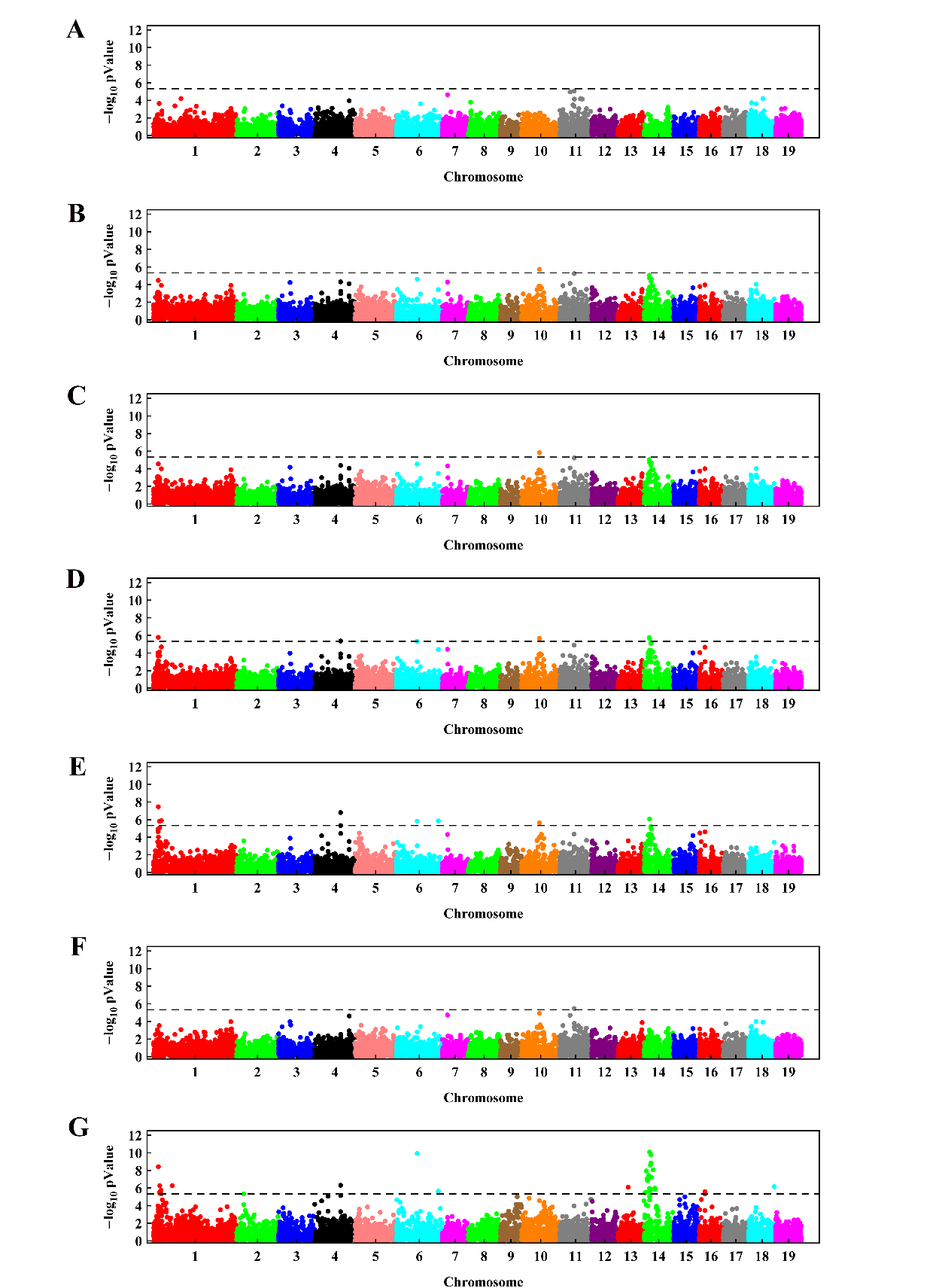


**S2 Fig.** Manhattan plots of the association analyses without genomic control for each univariate trait of L (A), W (B), W31 (C), W21 (D), W32 (E), A (F), and the ratio of L to W (G) across the 19 chromosomes of the reference genome of *P. trichocarpa*. The horizontal dashed line indicates the genome-wide significant threshold of 5.33, a base 10 logarithm of *p-*value based on the Bonferroni correction at the 0.05 significant level.
