# Supplementary material for: Multivariate genome-wide association study of leaf shape in a Populus deltoides and P. simonii F1 pedigree
Source: PLoS One. 2021 Oct 28;16(10):e0259278. doi: 10.1371/journal.pone.0259278 (PMC8553126; doi:10.1371/journal.pone.0259278)
Supplement: S1 Table — (DOCX) [file pone.0259278.s003.docx]

**S1 Table** The RADseq data information for the 2 parents and 163 progeny in the F_1_ hybrid population of *Populus deltoides* and *Populus simonii*.

| Parent/Progeny | Run | Spots | Bases | FR Length | RR Length | Sample Name |
| --- | --- | --- | --- | --- | --- | --- |
| P1 | SRR1785852 | 12949974 | 2589994800 | 100 | 100 | Maternal I-69 |
| P2 | SRR1777735 | 14024713 | 2804942600 | 100 | 100 | Paternal L-3 |
| P1 | SRR1795739 | 21619787 | 3718603364 | 82 | 90 | Maternal I-69 |
| P2 | SRR1795738 | 57159139 | 9912975139 | 82-85 | 90 | Paternal L-3 |
| 1 | SRR8374961 | 63361805 | 18628370670 | 144 | 150 | BGI3-0 |
| 2 | SRR8374968 | 41178453 | 12106465182 | 144 | 150 | BGI3-1 |
| 3 | SRR8377821 | 13512784 | 3999784064 | 145 | 151 | MBC3-103 |
| 4 | SRR8374963 | 27139454 | 7978999476 | 144 | 150 | BGI3-104 |
| 5 | SRR8374962 | 63026585 | 18529815990 | 144 | 150 | BGI3-107 |
| 6 | SRR8377820 | 9933855 | 2940421080 | 145 | 151 | MBC3-108 |
| 7 | SRR8374965 | 71980820 | 21306322720 | 146 | 150 | BGI3-109 |
| 8 | SRR8374967 | 50050296 | 14814887616 | 146 | 150 | BGI3-11 |
| 9 | SRR8374964 | 70592027 | 20895239992 | 146 | 150 | BGI3-110 |
| 10 | SRR8374966 | 65419981 | 19364314376 | 146 | 150 | BGI3-113 |
| 11 | SRR8379395 | 9076463 | 2686633048 | 146 | 150 | GPB3-116 |
| 12 | SRR8379396 | 12441410 | 3682657360 | 146 | 150 | GPB3-117 |
| 13 | SRR8374955 | 72345638 | 21414308848 | 146 | 150 | BGI3-12 |
| 14 | SRR8379422 | 8499265 | 2498873598 | 145 | 150 | GPB3-121 |
| 15 | SRR8379399 | 10030776 | 2959078920 | 145 | 150 | GPB3-122 |
| 16 | SRR8374954 | 68095630 | 19952019590 | 143 | 150 | BGI3-124 |
| 17 | SRR8379401 | 9239306 | 2707116658 | 143 | 150 | GPB3-128 |
| 18 | SRR8379403 | 10668186 | 3125778498 | 143 | 150 | GPB3-133 |
| 19 | SRR8377824 | 9863331 | 2939272638 | 147 | 151 | MBC3-14 |
| 20 | SRR8377799 | 12722122 | 3753025990 | 144 | 151 | MBC3-152 |
| 21 | SRR8377802 | 12290765 | 3625775675 | 144 | 151 | MBC3-153 |
| 22 | SRR8374946 | 65797912 | 19344586128 | 144 | 150 | BGI3-157 |
| 23 | SRR8377803 | 10350160 | 3073997520 | 146 | 151 | MBC3-16 |
| 24 | SRR8377804 | 9494350 | 2800833250 | 144 | 151 | MBC3-160 |
| 25 | SRR8374971 | 81018726 | 23981542896 | 146 | 150 | BGI3-167 |
| 26 | SRR8374976 | 69777678 | 20654192688 | 146 | 150 | BGI3-171 |
| 27 | SRR8374977 | 79725633 | 23519061735 | 145 | 150 | BGI3-172 |
| 28 | SRR8377797 | 13354877 | 3926333838 | 143 | 151 | MBC3-176 |
| 29 | SRR8374975 | 70354200 | 20613780600 | 143 | 150 | BGI3-177 |
| 30 | SRR8377793 | 13199805 | 3880742670 | 143 | 151 | MBC3-178 |
| 31 | SRR8374978 | 72641827 | 21284055311 | 143 | 150 | BGI3-179 |
| 32 | SRR8374979 | 81313562 | 23743560104 | 142 | 150 | BGI3-180 |
| 33 | SRR8374944 | 73245827 | 21387781484 | 142 | 150 | BGI3-185 |
| 34 | SRR8377794 | 14395855 | 4232381370 | 143 | 151 | MBC3-186 |
| 35 | SRR8374988 | 72068085 | 21332153160 | 146 | 150 | BGI3-19 |
| 36 | SRR8377796 | 11196338 | 3302919710 | 144 | 151 | MBC3-23 |
| 37 | SRR8374951 | 73550777 | 21697479215 | 145 | 150 | BGI3-25 |
| 38 | SRR8377789 | 8480599 | 2501776705 | 144 | 151 | MBC3-28 |
| 39 | SRR8377790 | 9963582 | 2949220272 | 145 | 151 | MBC3-3 |
| 40 | SRR8379405 | 8964334 | 2635514196 | 144 | 150 | GPB3-30 |
| 41 | SRR8377791 | 10786267 | 3181948765 | 144 | 151 | MBC3-32 |
| 42 | SRR8377792 | 11907277 | 3500739438 | 143 | 151 | MBC3-34 |
| 43 | SRR8379406 | 7921579 | 2344787384 | 146 | 150 | GPB3-38 |
| 44 | SRR8377788 | 8784940 | 2582772360 | 143 | 151 | MBC3-39 |
| 45 | SRR8377809 | 12549912 | 3714773952 | 145 | 151 | MBC3-4 |
| 46 | SRR8377810 | 10177394 | 2992153836 | 143 | 151 | MBC3-41 |
| 47 | SRR8379408 | 7741508 | 2291486368 | 146 | 150 | GPB3-43 |
| 48 | SRR8377808 | 12964368 | 3811524192 | 143 | 151 | MBC3-46 |
| 49 | SRR8374943 | 65336031 | 19143457083 | 143 | 150 | BGI3-47 |
| 50 | SRR8379424 | 13150758 | 3866355519 | 144 | 150 | GPB3-50 |
| 51 | SRR8379423 | 8247661 | 2433059995 | 145 | 150 | GPB3-51 |
| 52 | SRR8379426 | 7661859 | 2244924687 | 143 | 150 | GPB3-52 |
| 53 | SRR8374987 | 75441379 | 22104324047 | 143 | 150 | BGI3-53 |
| 54 | SRR8374980 | 52180586 | 15236731112 | 142 | 150 | BGI3-54 |
| 55 | SRR8374982 | 78931083 | 23205738402 | 144 | 150 | BGI3-6 |
| 56 | SRR8374981 | 56840061 | 16597297812 | 142 | 150 | BGI3-61 |
| 57 | SRR8379430 | 7076595 | 2066365740 | 142 | 150 | GPB3-69 |
| 58 | SRR8377812 | 24132485 | 7143215560 | 145 | 151 | MBC3-7 |
| 59 | SRR8374983 | 49289392 | 14392502464 | 142 | 150 | BGI3-70 |
| 60 | SRR8377813 | 9338401 | 2745489894 | 143 | 151 | MBC3-71 |
| 61 | SRR8379432 | 8552418 | 2497306056 | 142 | 150 | GPB3-74 |
| 62 | SRR8379431 | 7469121 | 2180983332 | 142 | 150 | GPB3-76 |
| 63 | SRR8379393 | 7987400 | 2332320800 | 142 | 150 | GPB3-78 |
| 64 | SRR8374984 | 54548996 | 15928306832 | 142 | 150 | BGI3-83 |
| 65 | SRR8379404 | 12957757 | 3809580558 | 144 | 150 | GPB3-84 |
| 66 | SRR8379420 | 11191349 | 3290256606 | 144 | 150 | GPB3-86 |
| 67 | SRR8379421 | 8955839 | 2633016666 | 144 | 150 | GPB3-87 |
| 68 | SRR8374985 | 62119339 | 18138846988 | 142 | 150 | BGI3-89 |
| 69 | SRR8377811 | 10315296 | 3073958208 | 147 | 151 | MBC3-9 |
| 70 | SRR8374986 | 63876989 | 18652080788 | 142 | 150 | BGI3-91 |
| 71 | SRR1804145 | 14161140 | 2478199500 | 85 | 90 | BGI-167/A10-1 |
| 72 | SRR1793215 | 3940709 | 788141800 | 100 | 100 | NHS068-48_L7/A10-3 |
| 73 | SRR1800180 | 6359316 | 1100161668 | 83 | 90 | BGI-140/A11-1 |
| 74 | SRR1804594 | 5412652 | 947214100 | 85 | 90 | BGI-173/A11-2 |
| 75 | SRR1795762 | 7628851 | 1327420074 | 84 | 90 | BGI-110/A12-1 |
| 76 | SRR1797643 | 5910281 | 1016568332 | 82 | 90 | BGI-115/A12-2 |
| 77 | SRR1806734 | 9835561 | 1691716492 | 82 | 90 | BGI-97/A13-2 |
| 78 | SRR1797682 | 6747398 | 1174047252 | 84 | 90 | BGI-124/A13-3 |
| 79 | SRR1802683 | 7152932 | 1230304304 | 82 | 90 | BGI-150/A14-3 |
| 80 | SRR1797657 | 5529216 | 951025152 | 82 | 90 | BGI-118/A15-2 |
| 81 | SRR1802686 | 4861270 | 836138440 | 82 | 90 | BGI-152/A16-1 |
| 82 | SRR1797678 | 6385274 | 1098267128 | 82 | 90 | BGI-123/A17-1 |
| 83 | SRR1797675 | 7217713 | 1255882062 | 84 | 90 | BGI-122/A18-2 |
| 84 | SRR1785870 | 5965531 | 1193106200 | 100 | 100 | NHS065-47_L3/A2-1 |
| 85 | SRR1806735 | 4819682 | 828985304 | 82 | 90 | BGI-98/A21-2 |
| 86 | SRR1802722 | 7448669 | 1296068406 | 84 | 90 | BGI-156/A21-3 |
| 87 | SRR1789493 | 2848728 | 569745600 | 100 | 100 | NHS066-42_L4/A2-2 |
| 88 | SRR1806730 | 8989630 | 1555205990 | 83 | 90 | BGI-93/A23-2 |
| 89 | SRR1784920 | 3982990 | 796598000 | 100 | 100 | NHS064-51_L2/A24-1 |
| 90 | SRR1793217 | 4867018 | 973403600 | 100 | 100 | NHS068-4_L7/A24-3 |
| 91 | SRR1802689 | 2736413 | 476135862 | 84 | 90 | BGI-154/A25-1 |
| 92 | SRR1802678 | 7103105 | 1221734060 | 82 | 90 | BGI-147/A25-3 |
| 93 | SRR1784928 | 4999731 | 999946200 | 100 | 100 | NHS064-55_L2/A26-1 |
| 94 | SRR1806727 | 20593690 | 3562708370 | 83 | 90 | BGI-89/A26-3 |
| 95 | SRR1806729 | 8500661 | 1487615675 | 85 | 90 | BGI-91/A27-1 |
| 96 | SRR1784922 | 3297179 | 659435800 | 100 | 100 | NHS064-52_L2/A31-2 |
| 97 | SRR1797636 | 6769497 | 1164353484 | 82 | 90 | BGI-114/A3-3 |
| 98 | SRR1797651 | 5841156 | 1004678832 | 82 | 90 | BGI-117/A4-2 |
| 99 | SRR1789501 | 3745887 | 749177400 | 100 | 100 | NHS066-4_L4/A4-3 |
| 100 | SRR1793222 | 5208880 | 1041776000 | 100 | 100 | NHS068-54_L7/A5-3 |
| 101 | SRR1783058 | 4979587 | 995917400 | 100 | 100 | NHS058-53_L4/A7-3 |
| 102 | SRR1804598 | 5454758 | 943673134 | 83 | 90 | BGI-177/A8-2 |
| 103 | SRR1785861 | 7202122 | 1440424400 | 100 | 100 | NHS065-43_L3/A8-3 |
| 104 | SRR1802721 | 2917815 | 507699810 | 84 | 90 | BGI-155/B1-1 |
| 105 | SRR1783033 | 5691595 | 1138319000 | 100 | 100 | NHS058-45_L4/B11-3 |
| 106 | SRR1804589 | 6193714 | 1065318808 | 82 | 90 | BGI-169/B1-2 |
| 107 | SRR1784913 | 3004933 | 600986600 | 100 | 100 | NHS064-48_L2/B12-1 |
| 108 | SRR1789498 | 3992798 | 798559600 | 100 | 100 | NHS066-47_L4/B15-3 |
| 109 | SRR1793223 | 3038826 | 607765200 | 100 | 100 | NHS068-55_L7/B17-1 |
| 110 | SRR1785862 | 5175211 | 1035042200 | 100 | 100 | NHS065-44_L3/B17-2 |
| 111 | SRR1791553 | 4219343 | 843868600 | 100 | 100 | NHS067-44_L6/B23-3 |
| 112 | SRR1797662 | 7705856 | 1340818944 | 84 | 90 | BGI-11/B29-2 |
| 113 | SRR1805367 | 6426964 | 1118291736 | 84 | 90 | BGI-52/B30-1 |
| 114 | SRR1805572 | 6383291 | 1097926052 | 82 | 90 | BGI-58/B30-2 |
| 115 | SRR1806717 | 4483949 | 780207126 | 84 | 90 | BGI-76/B30-3 |
| 116 | SRR1804601 | 7584695 | 1334906320 | 86 | 90 | BGI-18/B31-1 |
| 117 | SRR1804602 | 4324495 | 756786625 | 85 | 90 | BGI-19/B34-1 |
| 118 | SRR1805110 | 8432336 | 1450361792 | 82 | 90 | BGI-42/B36-1 |
| 119 | SRR1806722 | 8569513 | 1508234288 | 86 | 90 | BGI-82/B5-2 |
| 120 | SRR1806732 | 6410545 | 1102613740 | 82 | 90 | BGI-95/B5-3 |
| 121 | SRR1806725 | 7266618 | 1271658150 | 85 | 90 | BGI-84/B7-2 |
| 122 | SRR1802685 | 6631661 | 1140645692 | 82 | 90 | BGI-151/B9-2 |
| 123 | SRR1785892 | 4097434 | 819486800 | 100 | 100 | NHS065-52_L3/B9-3 |
| 124 | SRR1783234 | 4768037 | 953607400 | 100 | 100 | NHS063-57_L1/C10-1 |
| 125 | SRR1805696 | 5896953 | 1037863728 | 86 | 90 | BGI-69/C1-3 |
| 126 | SRR1783150 | 4960771 | 992154200 | 100 | 100 | NHS063-45_L1/C20-1 |
| 127 | SRR1805109 | 6953147 | 1195941284 | 82 | 90 | BGI-41/C2-1 |
| 128 | SRR1783152 | 4772799 | 954559800 | 100 | 100 | NHS063-47_L1/C23-2 |
| 129 | SRR1785873 | 2771889 | 554377800 | 100 | 100 | NHS065-48_L3/C23-3 |
| 130 | SRR1805036 | 4821566 | 838952484 | 84 | 90 | BGI-27/C25-3 |
| 131 | SRR1783149 | 3736224 | 747244800 | 100 | 100 | NHS063-44_L1/C28-1 |
| 132 | SRR1802728 | 6679886 | 1175659936 | 86 | 90 | BGI-15/C3-1 |
| 133 | SRR1804713 | 7163320 | 1239254360 | 83 | 90 | BGI-26/C3-3 |
| 134 | SRR1805569 | 4499445 | 773904540 | 82 | 90 | BGI-57/C33-3 |
| 135 | SRR1804711 | 7820450 | 1352937850 | 83 | 90 | BGI-24/C36-1 |
| 136 | SRR1793214 | 3920725 | 784145000 | 100 | 100 | NHS068-47_L7/C36-3 |
| 137 | SRR1783231 | 3678095 | 735619000 | 100 | 100 | NHS063-54_L1/C37-2 |
| 138 | SRR1806718 | 4622230 | 813512480 | 86 | 90 | BGI-77/C5-3 |
| 139 | SRR1783063 | 4219727 | 843945400 | 100 | 100 | NHS058-56_L4/D1-3 |
| 140 | SRR1805104 | 7265734 | 1249706248 | 82 | 90 | BGI-38/D14-2 |
| 141 | SRR1784932 | 6465741 | 1293148200 | 100 | 100 | NHS064-58_L2/D17-1 |
| 142 | SRR1795755 | 7500914 | 1290157208 | 82 | 90 | BGI-104/D19-1 |
| 143 | SRR1804142 | 12412472 | 2172182600 | 85 | 90 | BGI-164/D19-2 |
| 144 | SRR1800145 | 7399169 | 1302253744 | 86 | 90 | BGI-130/D20-2 |
| 145 | SRR1797660 | 6691046 | 1164242004 | 84 | 90 | BGI-119/D21-1 |
| 146 | SRR1784931 | 4265382 | 853076400 | 100 | 100 | NHS064-57_L2/D22-2 |
| 147 | SRR1797673 | 5740433 | 998835342 | 84 | 90 | BGI-120/D22-3 |
| 148 | SRR1783048 | 4545402 | 909080400 | 100 | 100 | NHS058-4_L4/D2-3 |
| 149 | SRR1785901 | 4316703 | 863340600 | 100 | 100 | NHS065-56_L3/D24-3 |
| 150 | SRR1797633 | 5152198 | 886178056 | 82 | 90 | BGI-113/D25-2 |
| 151 | SRR1800042 | 7284298 | 1282036448 | 86 | 90 | BGI-127/D25-3 |
| 152 | SRR1789510 | 5224309 | 1044861800 | 100 | 100 | NHS066-58_L4/D26-1 |
| 153 | SRR1800177 | 6929752 | 1198847096 | 83 | 90 | BGI-139/D27-3 |
| 154 | SRR1783025 | 5371233 | 1074246600 | 100 | 100 | NHS058-43_L4/D28-2 |
| 155 | SRR1789500 | 3453940 | 690788000 | 100 | 100 | NHS066-49_L4/D29-2 |
| 156 | SRR1791561 | 3314507 | 662901400 | 100 | 100 | NHS067-51_L6/D4-3 |
| 157 | SRR1805113 | 6434509 | 1113170057 | 83 | 90 | BGI-46/D5-2 |
| 158 | SRR1793232 | 10909376 | 2181875200 | 100 | 100 | NHS069-45_L8/D5-3 |
| 159 | SRR1793219 | 3578654 | 715730800 | 100 | 100 | NHS068-51_L7/D6-2 |
| 160 | SRR1784904 | 3759691 | 751938200 | 100 | 100 | NHS064-41_L2/D6-3 |
| 161 | SRR1805117 | 6036923 | 1038350756 | 82 | 90 | BGI-49/D7-3 |
| 162 | SRR1805597 | 2886219 | 496429668 | 82 | 90 | BGI-5/D8-1 |
| 163 | SRR1789497 | 3910016 | 782003200 | 100 | 100 | NHS066-46_L4/D8-2 |
