# Supplementary material for: Multivariate genome-wide association study of leaf shape in a Populus deltoides and P. simonii F1 pedigree
Source: PLoS One. 2021 Oct 28;16(10):e0259278. doi: 10.1371/journal.pone.0259278 (PMC8553126; doi:10.1371/journal.pone.0259278)
Supplement: S4 Table — (DOCX) [file pone.0259278.s006.docx]

**S4 Table** Correlation coefficients among the leaf traits of L, W, W31, W21, W32, A, and the ratio of L to W in the randomized complete block design derived from the F1 progeny of *Populus deltoides* $\times$ *Populus simonii*.

|  | L | W | W1/3 | W1/2 | W2/3 | A |
| --- | --- | --- | --- | --- | --- | --- |
| W | 0.9214^**^ |  |  |  |  |  |
| W1/3 | 0.9219^**^ | 0.9989^**^ |  |  |  |  |
| W1/2 | 0.8921^**^ | 0.9905^**^ | 0.9897^**^ |  |  |  |
| W2/3 | 0.8137^**^ | 0.9506^**^ | 0.9480^**^ | 0.9774^**^ |  |  |
| A | 0.9425^**^ | 0.9728^**^ | 0.9697^**^ | 0.9573^**^ | 0.9182^**^ |  |
| L/W | -0.2389^**^ | -0.5800^**^ | -0.5773^**^ | -0.6160^**^ | -0.6704^**^ | -0.4441^**^ |

_­­_L: leaf length; W: maximum leaf width; W1/3: leaf width at one-third length; W1/2: leaf width at half length; W2/3: leaf width at two-thirds length; A: area; L/W: the ratio of the leaf length to the maximum width; ^**^$: P<0.0001$.
