# Supplementary material for: Multivariate genome-wide association study of leaf shape in a Populus deltoides and P. simonii F1 pedigree
Source: PLoS One. 2021 Oct 28;16(10):e0259278. doi: 10.1371/journal.pone.0259278 (PMC8553126; doi:10.1371/journal.pone.0259278)
Supplement: S5 Table — (DOCX) [file pone.0259278.s007.docx]

**S5 Table** Analysis of variance for the leaf parameters of L, W, W1/3, W1/2, W2/3, and Area in the randomized complete block experiment derived from the F_1_ progeny of *Populus deltoides* × *Populus simonii*.

| Trait | Source | DF | MS | F | *P* value |
| --- | --- | --- | --- | --- | --- |
| L | Block | 2 | 21715.404 | 53.669 | 0.000 |
|  | Clone | 162 | 2330.908 | 5.761 | 0.000 |
|  | Error | 2079 | 404.621 |  |  |
|  |  |  |  |  |  |
| W | Block | 2 | 18570.688 | 56.800 | 0.000 |
|  | Clone | 162 | 2211.536 | 6.764 | 0.000 |
|  | Error | 2079 | 326.951 |  |  |
|  |  |  |  |  |  |
| W1/3 | Block | 2 | 18537.069 | 56.227 | 0.000 |
|  | Clone | 162 | 2172.801 | 6.591 | 0.000 |
|  | Error | 2079 | 329.682 |  |  |
|  |  |  |  |  |  |
| W1/2 | Block | 2 | 13789.192 | 51.401 | 0.000 |
|  | Clone | 162 | 1829.192 | 6.818 | 0.000 |
|  | Error | 2079 | 268.272 |  |  |
|  |  |  |  |  |  |
| W2/3 | Block | 2 | 7475.937 | 42.216 | 0.000 |
|  | Clone | 162 | 1331.362 | 7.518 | 0.000 |
|  | Error | 2079 | 177.088 |  |  |
|  |  |  |  |  |  |
| Area | Block | 2 | 328308011.6 | 52.088 | 0.000 |
|  | Clone | 162 | 47137504.67 | 7.479 | 0.000 |
|  | Error | 2079 | 6302948.369 |  |  |
