# Supplementary material for: Multivariate genome-wide association study of leaf shape in a Populus deltoides and P. simonii F1 pedigree
Source: PLoS One. 2021 Oct 28;16(10):e0259278. doi: 10.1371/journal.pone.0259278 (PMC8553126; doi:10.1371/journal.pone.0259278)
Supplement: S6 Table — (DOCX) [file pone.0259278.s008.docx]

**S6 Table**　Canonical correlation coefficients among the leaf length, widths, area, the length/width ratio, and polar radii in the randomized complete block design derived from the F1 progeny of *Populus deltoides* $\times$ *Populus simonii*.

| Traits | RD360 | RD61 | RD16 | RD11 | RD09 | RD06 |
| --- | --- | --- | --- | --- | --- | --- |
| L | 0.9994^**^ | 0.9988^**^ | 0.9988^**^ | 0.9987^**^ | 0.9988^**^ | 0.9987^**^ |
| W | 0.9998^**^ | 0.9988^**^ | 0.9986^**^ | 0.9985^**^ | 0.9985^**^ | 0.9983^**^ |
| W1/3 | 0.9997^**^ | 0.9981^**^ | 0.9978^**^ | 0.9977^**^ | 0.9976^**^ | 0.9975^**^ |
| W1/2 | 0.9996^**^ | 0.9983^**^ | 0.9982^**^ | 0.9980^**^ | 0.9979^**^ | 0.9977^**^ |
| W2/3 | 0.9979^**^ | 0.9958^**^ | 0.9953^**^ | 0.9949^**^ | 0.9949^**^ | 0.9934^**^ |
| Area | 0.9919^**^ | 0.9873^**^ | 0.9868^**^ | 0.9868^**^ | 0.9865^**^ | 0.9861^**^ |
| Ratio | 0.9628^**^ | 0.9463^**^ | 0.9445^**^ | 0.9439^**^ | 0.9434^**^ | 0.9426^**^ |
| LWs | 0.9999^**^ | 0.9998^**^ | 0.9997^**^ | 0.9997^**^ | 0.9997^**^ | 0.9996^**^ |

_­­_L: leaf length; W: maximum leaf width; W1/3: leaf width at one-third length; W1/2: leaf width at half length; W2/3: leaf width at two-thirds length; Ratio: the ratio of the leaf length to the maximum width; LWs: L, W, W31, W21, and W32; RD360: the 360 regular leaf polar radii; RD61: the 61 regular leaf polar radii on the right side; the 16 regular leaf polar radii on the right side; RD11: the 11 regular leaf polar radii on the right side; RD09: the 9 regular leaf polar radii on the right side; RD06: the 6 regular leaf polar radii on the right side; ^**^$: P<0.0001$.
