# Supplementary material for: Multivariate genome-wide association study of leaf shape in a Populus deltoides and P. simonii F1 pedigree
Source: PLoS One. 2021 Oct 28;16(10):e0259278. doi: 10.1371/journal.pone.0259278 (PMC8553126; doi:10.1371/journal.pone.0259278)
Supplement: S7 Table — (DOCX) [file pone.0259278.s009.docx]

**S7 Table** Correlation coefficients between the first principal component of different radius datasets and the leaf length, different widths, or area in the randomized complete block design derived from the F1 progeny of *Populus deltoides* $\times$ *Populus simonii*.

|  | L | W | W1/3 | W1/2 | W2/3 | A |
| --- | --- | --- | --- | --- | --- | --- |
| RD360 | 0.9611^**^ | 0.9890^**^ | 0.9880^**^ | 0.9764^**^ | 0.9325^**^ | 0.9857^**^ |
| RD61 | 0.9622^**^ | 0.9883^**^ | 0.9873^**^ | 0.9754^**^ | 0.9308^**^ | 0.9853^**^ |
| RD16 | 0.9659^**^ | 0.9868^**^ | 0.9859^**^ | 0.9732^**^ | 0.9269^**^ | 0.9849^**^ |
| RD11 | 0.9683^**^ | 0.9857^**^ | 0.9849^**^ | 0.9716^**^ | 0.9240^**^ | 0.9845^**^ |
| RD09 | 0.9725^**^ | 0.9828^**^ | 0.9820^**^ | 0.9672^**^ | 0.9173^**^ | 0.9839^**^ |
| RD06 | 0.9746^**^ | 0.9821^**^ | 0.9814^**^ | 0.9664^**^ | 0.9150^**^ | 0.9827^**^ |

_­­_RD360: the 360 regular leaf polar radii; RD61: the 61 regular leaf polar radii on the right side; RD16: the 16 regular leaf polar radii on the right side; RD11: the 11 regular leaf polar radii on the right side; RD09: the 9 regular leaf polar radii on the right side; RD06: the 6 regular leaf polar radii on the right side; L: leaf length; W: maximum leaf width; W1/3: leaf width at one-third length; W1/2: leaf width at half length; W2/3: leaf width at two-thirds length; A: area; ^**^$: P<0.0001$.
