# Supplementary material for: Multivariate genome-wide association study of leaf shape in a Populus deltoides and P. simonii F1 pedigree
Source: PLoS One. 2021 Oct 28;16(10):e0259278. doi: 10.1371/journal.pone.0259278 (PMC8553126; doi:10.1371/journal.pone.0259278)
Supplement: S8 Table — (DOCX) [file pone.0259278.s010.docx]

**S8 Table** Summary of significant SNPs associated to each trait of the four different leaf widths and area without genomic control.

| Trait | Chromosome | Position | Segregation Type | *P*-Value | -log(*P-*Value) | PVE (%) |
| --- | --- | --- | --- | --- | --- | --- |
| Width | 10 | 10,669,990 | *aa*×*ab* | 1.82E-6 | 5.74 | 1.01 |
| 1/3Width | 10 | 10,669,990 | *aa*×*ab* | 1.49E-6 | 5.83 | 1.03 |
| 1/2Width | 1 | 2,742,828 | *aa*×*ab* | 1.64E-6 | 5.78 | 1.02 |
|  | 4 | 15,591,673 | *aa*×*ab* | 4.30E-6 | 5.37 | 0.94 |
|  | 6 | 12,471,729 | *ab*×*aa* | 4.65E-6 | 5.33 | 0.93 |
|  | 10 | 10,669,990 | *aa*×*ab* | 2.19E-6 | 5.66 | 1.00 |
|  | 14 | 3,280,114 | *ab*×*aa* | 1.69E-6 | 5.77 | 1.02 |
|  | 14 | 3,466,838 | *ab*×*aa* | 2.28E-6 | 5.64 | 0.99 |
| 2/3Width | 1 | 2,742,828 | *ab*×*aa* | 3.41E-8 | 7.47 | 1.35 |
|  | 1 | 3,483,919 | *ab*×*aa* | 1.51E-6 | 5.82 | 1.03 |
|  | 1 | 4,571,250 | *ab*×*aa* | 1.27E-6 | 5.90 | 1.04 |
|  | 4 | 15,591,673 | *ab*×*aa* | 1.57E-7 | 6.80 | 1.22 |
|  | 6 | 12471,729 | *ab*×*aa* | 1.50E-6 | 5.82 | 1.03 |
|  | 6 | 25,163,982 | *ab*×*aa* | 1.41E-6 | 5.85 | 1.03 |
|  | 10 | 10,669,990 | *aa*×*ab* | 2.27E-6 | 5.64 | 0.99 |
|  | 14 | 3,466,838 | *ab*×*aa* | 8.51E-7 | 6.07 | 1.08 |
| Area | 11 | 8,744,108 | *aa*×*ab* | 3.39E-6 | 5.47 | 0.96 |
