# Supplementary material for: Multivariate genome-wide association study of leaf shape in a Populus deltoides and P. simonii F1 pedigree
Source: PLoS One. 2021 Oct 28;16(10):e0259278. doi: 10.1371/journal.pone.0259278 (PMC8553126; doi:10.1371/journal.pone.0259278)
Supplement: S9 Table — (DOCX) [file pone.0259278.s011.docx]

**S9 Table** Summary of significant SNPs associated to the ratio of the leaf length to the maximum width without genomic control.

| Chromosome | Position | Segregation Type | *P*-Value | -log(*P-*Value) | PVE (%) |
| --- | --- | --- | --- | --- | --- |
| 1 | 2799619 | *ab*×*aa* | 3.77E-9 | 8.42 | 1.54 |
|  | 3483919 | *ab*×*aa* | 2.91E-6 | 5.54 | 0.97 |
|  | 3620732 | *ab*×*aa* | 3.7E-6 | 5.43 | 0.95 |
|  | 3631642 | *ab*×*aa* | 5.36E-7 | 6.27 | 1.12 |
|  | 4255086 | *ab*×*aa* | 1.78E-6 | 5.75 | 1.01 |
|  | 4573250 | *ab*×*aa* | 4.58E-6 | 5.34 | 0.93 |
| 1 | 11059517 | *ab*×*aa* | 5.37E-7 | 6.27 | 1.12 |
| 2 | 4266925 | *aa*×*ab* | 4.55E-6 | 5.34 | 0.93 |
| 4 | 15591673 | *ab*×*aa* | 4.88E-7 | 6.31 | 1.12 |
| 6 | 12471729 | *ab*×*aa* | 1.17E-10 | 9.93 | 1.84 |
| 6 | 25163980 | *ab*×*aa* | 2.22E-6 | 5.65 | 0.99 |
| 13 | 6260813 | *ab*×*aa* | 8.26E-7 | 6.08 | 1.08 |
| 14 | 749516 | *ab*×*aa* | 3.39E-6 | 5.47 | 0.96 |
|  | 1270652 | *ab*×*aa* | 2.97E-6 | 5.53 | 0.97 |
|  | 1485873 | *ab*×*aa* | 1.14E-8 | 7.94 | 1.44 |
|  | 1814655 | *ab*×*aa* | 9.73E-8 | 7.01 | 1.26 |
| 14 | 2326098 | *ab*×*aa* | 1.47E-7 | 6.83 | 1.23 |
|  | 2636609 | *ab*×*aa* | 6.76E-8 | 7.17 | 1.29 |
|  | 2854827 | *ab*×*aa* | 3.73E-8 | 7.43 | 1.34 |
|  | 3370505 | *ab*×*aa* | 1.13E-6 | 5.95 | 1.05 |
|  | 3467027 | *ab*×*aa* | 8.38E-11 | 10.08 | 1.87 |
| 14 | 3910143 | *ab*×*aa* | 2.56E-9 | 8.59 | 1.57 |
|  | 4014340 | *ab*×*aa* | 1.43E-6 | 5.84 | 1.03 |
|  | 4210289 | *ab*×*aa* | 1.6E-10 | 9.79 | 1.81 |
|  | 4309715 | *ab*×*aa* | 1.39E-9 | 8.86 | 1.62 |
|  | 4316988 | *ab*×*aa* | 5.73E-8 | 7.24 | 1.31 |
|  | 4564789 | *ab*×*aa* | 6.12E-8 | 7.21 | 1.3 |
| 14 | 5494986 | *ab*×*cc* | 8.68E-9 | 8.06 | 1.47 |
| 14 | 6606004 | *ab*×*aa* | 1.06E-6 | 5.98 | 1.06 |
|  | 6956257 | *ab*×*aa* | 1.34E-6 | 5.87 | 1.04 |
| 16 | 3663208 | *ab*×*aa* | 4.39E-6 | 5.36 | 0.94 |
|  | 3666960 | *ab*×*aa* | 2.68E-6 | 5.57 | 0.98 |
| 18 | 15471331 | *ab*×*aa* | 6.99E-7 | 6.16 | 1.09 |
