# Supplementary material for: Multivariate genome-wide association study of leaf shape in a Populus deltoides and P. simonii F1 pedigree
Source: PLoS One. 2021 Oct 28;16(10):e0259278. doi: 10.1371/journal.pone.0259278 (PMC8553126; doi:10.1371/journal.pone.0259278)
Supplement: Attachment — Submitted filename: Response to Reviewers.docx [file pone.0259278.s014.docx]

**Response to Reviewers’ Comments:**

Reviewer #1: Overall the manuscript is clear and well written and the results support the conclusions. It presents an interesting analysis of leaf shape.

I have several specific points that need clarification.

1. The abstract is clear and the results are well presented but it doesn't say why poplar or why leaf shape. A sentance explaining this in the abstract would make it clearer because at present the reason for using poplar isn't really standing out.

RE: Thank you very much for your suggestion. We added a sentence in Abstract to address the importance of poplar and its leaf shape as follows: “Most poplar species are of great economic and ecological values and their leaf morphology can be a predictor for wood productivety and environment adaptation”.

2. Line 54 in the introduction- polygenic is a standard term and doesn't really need defining here

RE: Thanks. We deleted the words in the bracket as you suggested.

3. The QTL analysis needs more detail in the methods section.

RE: Thank you for this suggestion. After Line 140, we described the mvLMM for details in a single data form as follows:

| “ $y_{ijkl}=\mu_{l}+B_{il}+M_{jl}+G_{jl}+e_{ijkl}$ | (1) |
| --- | --- |

where $y_{ijkl}$ is the *l*th polar radius of the *k*th tree leaf of the *j*th clone in the *i*th block; $\mu_{l}$ is the overall mean of the *l*th polar radius; $B_{il}$ is the effect of the *i*th block; $M_{jl}$ is the genotype effect of the *j*th clone at any tested SNP; $G_{jl}$ is the polygenic background effect of the *j*th clone; and $e_{ijkl}$ is the residual effect. It is assumed that $B_{il}$ and $M_{jl}$ are fixed effects, while $G_{jl}$ and $e_{ijkl}$ are random effects with $G_{jl}\sim N(0, \sigma_{g_{l}}^{2})$, $e_{ijkl}\sim N(0, \sigma_{e_{l}}^{2})$ and $\mathrm{cov}(G_{jl}, e_{ijkl})=0$. In matrix form, model (1) can be written as …”.

4. Line 91 needs another look at the grammar:

RE: Thanks. We modified the sentence as “we identified many more SNPs significantly associated with leaf shape than those detected in previous studies”.

5. I know this is mentioned in the introduction but in the methods you could make it clear which is the male and female parent in your cross.

RE: Thank you very much for your suggestion. In Line 103, we changed “*P. deltoides* and *P. simonii*” to “the female *P. deltoides* and the male *P. simonii*”.

6. Line 153 a rather than an SNP

RE: Thanks. We modified “an SNP” as “any single SNP”.

7. Line 186 it isn't clear where the 2,244 samples came from. The methods state 3 blocks and 6 leaves per block

RE: Thanks for your comments. We inserted a sentence there to explain that there were a few missed samples in some plots as follows: “Some plots had missed samples due to the damage from pest, disease, poor rooting ability, or other unknown reasons”.

8. Line 239 The sentance needs rewriting, should it be -with few SNPs on chromosomes

RE: Thanks for pointing out this grammar error. We modified it as “with a few SNPs on chromosomes …”.

Reviewer #2: This manuscript described QTL mapping in poplar. A segregating mapping population was used as plant material, while a GWAS model was employed for data analysis. The result is interesting; however, I do have some concerns. The author should consider the below points.

1. QTL mapping was conducted with segregating population. Did the author considered the population structure (Q) and kinship (K)? How did the mvLMM work? I noticed the model Y= XB+ZG + E, it seems there is similar parameters of Q and K in this model, if so, how did the author calculate them?

RE: Thank you very much for your comments.

1) Because our samples were from a single full-sib family, the population was so simple that it is improper to apply the population structure (Q) model to the current GWAS. In fact, the mvLMM involved in the kinship matrix (K) with the relationship of A=2K, where A is the additive relationship matrix and the K was estimated from the genetic theory [38]. The relationship of A and K was clearly described in Lines 150-152.

2) Y= XB+ZG + E is the mvLMM model in matrix form, which is very complicated. However, in theory, it is more mature because it belongs to linear model and is widely applied. The F statistics was presented as Equation (3) for testing a single SNP, which can be calculated with the R package EMMREML (https://cran.r-project.org/web/packages/EMMREML). Please see Lines 171-174 for details.

2. The author investigated 100 genes for a significant SNPs, why 100? Usually, the physical distance would be used as a threshold.

RE: Thanks. We took the so-called proximate strategy to investigate the nearby genes of each significant SNP (Monclus et al. 2012; Geng et al. 2015; Su et al. 2017; Vanous et al. 2018). Those genes that have annotations related to the leaf growth and development were chosen as candidate genes, which were listed in Table 3. However, in literatures, there is no conclusion about how many nearby genes should be provided for possible candidates. We chose 100 nearby genes, expecting to find as many as possible candidate genes for a significant SNP. In order to clarify this point, we cited the 4 literatures in Line 177.

Similarly, the threshold of a physical distance could be used to investigate the nearby genes, but the number of candidate genes certainly varied along QTLs.

Monclus et al. 2012. Integrating genome annotation and QTL position to identify candidate genes for productivity, architecture and water-use efficiency in Populus spp. BMC Plant Biology 12:173 DOI 10.1186/1471-2229-12-173.

Geng et al. 2015. A genome-wide association study in catfish reveals the presence of functional hubs of related genes within QTLs for columnaris disease resistance. BMC Genomics 16:196 DOI 10.1186/s12864-015-1409-4.

Su et al. 2017. High density linkage map construction and mapping of yield trait QTLs in maize (Zea mays) using the genotyping-by-sequencing (GBS) technology. Frontiers In Plant Science 8 DOI 10.3389/fpls.2017.00706.

Vanous et al. 2018. Association mapping of flowering and height traits in germplasm enhancement of maize doubled haploid (GEM-DH) lines. Plant Genome 11(2):1-14 DOI 10.3835/plantgenome2017.09.0083.

3. The QTL results seemed not good. The author compared their results with some previous reports. But I found they missed a very similar study. In that study, P. deltoides and P. simonii were also used as parents for a F1 population. See https://doi.org/10.1007/s00425-018-2958-y, is there any overlapping regions between the 2 studies?

RE: Thanks for your comments. Yes, this literature was about QTL study for leaf shape, but it was based on linkage maps and no physical position information was available for the QTLs detected. In Lines 338-339, we wrote “S11 Table lists those significant SNPs associated with leaf shape in the current study and in three recent studies [17, 18, 26], excluding the previous QTL studies because no position information was available on the physical maps for those QTLs related to poplar leaf shape [11, 25, 27, 28]”. Nevertheless, we cited this literature there as reference 63.
