# Supplementary material for: Multivariate genome-wide association study of leaf shape in a Populus deltoides and P. simonii F1 pedigree
Source: PLoS One. 2021 Oct 28;16(10):e0259278. doi: 10.1371/journal.pone.0259278 (PMC8553126; doi:10.1371/journal.pone.0259278)
Supplement: Attachment — Submitted filename: Response to Reviewers 20211005.docx [file pone.0259278.s015.docx]

**Response to Reviewers’ Comments:**

Reviewer #1: All of the comments and suggestions that I made have now been addressed and clearly answered. The manuscript is now much improved after all comments and suggestions have been taken on board.

RE: Thank you very much for your positive comments.

Reviewer #2: In the last round of review, I asked 3 questions, but the author did not provide the answerers directly. The first question is about the calculation of parameters in the association model. Yes, the equation was provided in the M&M, however, how did you match your data to each parameter. For example, in the GWAS model, it is not right to use all SNPs for Q calculation, but all SNPs should be used for K calculation. So, how did you calculate A and K?

RE: Thank you very much for pointing out this issue again.

1) In this study, the clones were from a full-sib family. In theory, for any two clones, the coefficient of kinship (constituted to K matrix) is expected to be 0.25 (Loiselle et al. 1995; Lynch and Walsh 1998). This led to the relationship matrix (A=2K) with elements of ones on the diagonal and 0.5 elsewhere (Lynch and Walsh 1998; Bae et al. 2016). Here, it is unnecessary to calculate (in fact, to estimate) the matrix K with SNP data because the pedigree is fully known and each value in the K matrix is equal to 0.25 or 0.5. We think we have described this issue clearly and cited related literatures in Lines 152-159.

Certainly, in most GWAS studies where the natural population were used and the pedigrees were usually unknown, it need to estimate the kinship matrix with SNP data. However, from a statistical point of view, theoretical values are usually better than estimators because the accuracy of estimators depends not only on the completeness of sampling data but also on the estimate method.

2) Population structure (Q) was originally used in human GWAS for overcoming genomic inflation because the population was considered natural and more complicated. However, the Q method is not a “golden rule” for all GWAS, and sometimes, it failed to control genomic inflation (Kim 2019; https://doi.org/10.1101/647768). In the current study, we established the multivariate linear mixed model for GWAS of leaf shape according to the traditional randomized complete block design. Through adjusting the dimension of the regular polar radii data, we found that a moderate dimensional data can be used to successfully control genomic inflation and obtain a better result in finding significant SNPs associated to leaf shape. We think that our mvLMM for GWAS was successfully established and every parameter was described clearly. Moreover, the whole calculation for parameter estimates and significant hypothesis test was implemented with the R package EMMREML (https://cran.r-project.org/web/packages/EMMREML).

Bae, H., S. Monti, M. Montano, M.H. Steinberg, T.T. Perls et al., 2016 Learning Bayesian networks from correlated data. Sci Rep 6: 25156. https://doi.org/10.1038/srep25156

Loiselle, B.A., V.L. Sork, J. Nason, and C. Graham, 1995 Spatial genetic structure of a tropical understory shrub, Psychotria officinalis (Rubiaceae). American Journal of Botany 82: 1420-1425. https://doi.org/10.1002/j.1537-2197.1995.tb12679.x

Lynch, M., and B. Walsh, 1998 Genetics and Analysis of Quantitative Traits. Sunderland, MA, USA: Sinauer Associates, Inc.

The second question is for determination of candidate genes. Frankly, I don`t think 100 genes around the significant SNP were a right selection. In different regions of genome, the density of genes was different. In linkage-based QTL mapping, 95% confident interval was used, while LD length was used in GWAS. So, I think there should be also a strategy for you to define the candidate QTL region in the model you used. Yes, different strategies, including 100 genes, were used in some published paper, but this doesn`t mean all of them were reasonable.

RE: Thank you for pointing out this issue again. As you suggested, we modified the investigation of candidate genes with the method of LD analysis. But, we found the average LD length was estimated to be ~650 bp in our previous study (Chen et al., 2021; https://www.doi.org/10.1093/g3journal/jkaa053). It is so short and cannot be properly used as a range for candidate genes. Therefore, we considered genes within a LD block that contained a significant SNP for investigating candidate gene. The corresponding sections in M&M and Results as well as Table 3 and S10 Table were thoroughly modified.

The third question is for comparison between your results with a previous very similar study. The author didn`t perform the comparison because they could not find physical positions of SNP in that paper. Is that true? I am very sure there was physical position for each SNP. Also, the authors also provided physical position for each candidate region in that study.

RE: Thank you for pointing out this issue again and sorry for not carefully considering it in the previous manuscript. In fact, the accurate QTL position was described in genetic distance (cM), but not available in physical distance (bp). However, their physical position can be determined by flanking SNPs. Therefore, we performed the comparison by adding the work as you suggested. Please see the result in in S11 Table. Accordingly, the last paragraph in Discussion was modified.

I found the quality of the study is far above the quality of writing in this paper. Frankly, your work could be published some other high-reputational journals. The author should carefully revise their manuscript.

RE: Thank you very much for this positive comment. We carefully revised those paragraphs for investigating candidate genes and the comparison study with previous similar works.
